# The Therapeutic Effect of Shark Liver Oil in a Rat Model of Acetic Acid-Induced Ulcerative Colitis

**DOI:** 10.1155/2020/2419230

**Published:** 2020-10-21

**Authors:** Nastaran Samimi, Masood Sepehrimanesh, Omid Koohi-Hosseinabadi, Reza Homayounfar, Maral Mokhtari, Mojtaba Farjam

**Affiliations:** ^1^Noncommunicable Diseases Research Center, Fasa University of Medical Sciences, Fasa, Iran; ^2^Student Research Committee, Fasa University of Medical Sciences, Fasa, Iran; ^3^Gastroenterohepatology Research Center, Shiraz University of Medical Sciences, Shiraz, Iran; ^4^Central Research Laboratory, Shiraz University of Medical Sciences, Shiraz, Iran; ^5^Department of Pathology, School of Medicine, Shiraz University of Medical Sciences, Shiraz, Iran

## Abstract

Ulcerative colitis (UC) is one of the most well-known types of inflammatory bowel disease that manifests as recurrent inflammation of rectum and colon. The goal of this study is to evaluate the protective effects of shark liver oil (SLO) on acetic acid-induced ulcerative colitis in rats. Eighty induced UC rats were randomly divided into ten equal groups and received the following treatments for seven days: 1 ml of normal saline rectally, 1 ml of gel base (carboxymethyl cellulose) rectally, 10 mg/kg of Asacol rectally, 10 mg/kg of mesalazine orally, 5% gel form of SLO rectally, 10% gel form of SLO rectally, 200 mg of SLO orally, and 400 mg of SLO orally. We examined the oxidative stress indices, histopathological features, and body weight changes, as well as the function of the liver and kidneys at the end of treatment. Administration of 10% rectal and 400 mg oral SLO resulted in a significant weight gain. Also, glutathione peroxidase activity was significantly higher in 5% and 10% SLO-treated groups, and elevated superoxide dismutase activity in rats that received 5% SLO was observed compared to negative control and Asacol groups. While no significant changes were observed in most of the kidney and liver function markers, higher levels of aspartate aminotransferase were detected in the group that received 400 mg SLO orally compared to negative control and Asacol groups. Many histopathological signs of improvement were observed in mesalazine, Asacol, and SLO groups. There were no significant changes detected in the mean rank among different groups. Our data indicate that SLO supplementation could improve the amelioration of acetic acid-induced UC in rats due to its antioxidant effects.

## 1. Introduction

Ulcerative colitis (UC), as one of the main types of inflammatory bowel diseases (IBD), is a recurrent chronic disorder of rectal and colonic mucosa. The etiology of this disease is not entirely clear, but there are strong pieces of evidence that dysregulation of the immune response towards intraintestinal antigens could play a pivotal role in the initiation of UC and deterioration of patient's condition [1]. The incidence of UC has been the highest in Westernized nations since the past few decades, and it seems to be emerging in newly industrialized countries in Asia, South America, and the middle east [2]. This disease mostly initiates in the second and third decades of the patient's life and presents with diarrhea, abdominal cramps, rectal bleeding, tenesmus, and passage of mucus [3]. More importantly, UC increases the risk of colorectal cancer development in patients [4]. Although several environmental factors such as alteration of intestinal microbiota, exposure to antibiotics and air pollution, and smoking, as well as genetic factors, can predispose an individual to UC, there are firm shreds of evidence, emphasizing on the effect of the intestinal immune system dysregulation in the ulcerative colitis pathogenesis [5].

Current UC therapies include the application of 5-ASA agents, glucocorticoids, azathioprine, 6-mercaptopurine (6-MP), and biological agents [6, 7]. Many patients with UC have progressive courses that lead to hospitalization and surgical interventions [8]. Although new treatments, including biological agents, reduced the need to perform a surgery, the cost burden of such treatments is high for patients and healthcare systems [9, 10]. Therefore, physicians and scientists tend to use alternative medicine, including natural products, which have minimal side effects and on the other hand, reduce the cost burden of UC.

Shark liver oil (SLO) has been applied as a traditional marine natural product by Scandinavian people due to its possible therapeutic effects [11, 12]. SLO is rich in alkylglycerols and squalene and also contains n-3 polyunsaturated fatty acids (N-3 PUFA) in lower amounts [13, 14]. According to recent studies, SLO has multiple biological activities, including improving the immune system against infections by increasing neutrophil and macrophage activity [15, 16], stimulating hematopoiesis, erythropoiesis [17], antitumor and antimetastasis activities [18, 19], and alleviating the side effects of radiotherapy [20]. Due to lack of previous studies about therapeutic effects of SLO in UC and to find an alternative therapeutic agent for UC, we tried to evaluate the effects of SLO on acetic acid-induced UC in a rat model.

## 2. Materials and Methods

### 2.1. Ethical Statement

This study was approved by the Ethics Committee of Fasa University of Medical Sciences (registration number: IR.FUMS.REC.1395.89), and all procedures were along the lines with the Helsinki Declaration of 2008 [21].

### 2.2. Preparation of SLO

The liver of *Centrophorus ******granulosus* shark had been caught in the Persian Gulf during the autumn season and transferred on ice to the laboratory. The gallbladder was removed, and the rest of the liver was chopped and mixed. The mixed liver was weighed, and 500 gr of the mixture was placed in a cotton bag on an aluminum pan and cooked in a waterbath at 70–80°C for 30 minutes. Then, the cotton bag was hand-pressed to release the oil-water extract, and then, centrifuge (3000 rpm, 15 min, and room temperature) to separate the oil from the water [22]. The density of SLO was 0.9 gr/ml. To prepare the gel form of shark liver oil, 5 ml and 10 ml of the prepared shark liver oil were added to 95 ml and 90 ml of the gel base (carboxymethyl cellulose), respectively.

### 2.3. Animals

The Center of Comparative and Experimental Medicine, Shiraz University of Medical Sciences, Shiraz, Iran, provided 80 male Sprague–Dawley rats (300 ± 20 gr weight). Rats were housed in separated cages in a limited-access room with equal light and dark cycle, the ambient temperature of 22 ± 2°C, and 50% relative humidity. All animals fasted 24 hours before induction of UC. Therefore, their bowels were cleaned, and after the induction, standard chow diet and water were freely accessible for them.

### 2.4. Induction of UC

UC was imitated by the administration of 2 ml of 3% acetic acid, transrectally, under light anesthesia of ether inhalation using a 2 mm diameter cannula that inserted 8 cm proximal to the anus. After that, rats were positioned in a supine Trendelenburg position for 1 minute to avoid acetic acid leakage [23].

### 2.5. Experimental Design

After randomization, we allocated rats into eight groups (ten rats in each) as follows. All the animals received their treatments once a day for seven days.

- Group I (negative control 1) received 1 ml of normal saline rectally
- Group II (negative control 2) received 1 ml of gel base (carboxymethyl cellulose) rectally
- Group III (positive control 1) received 10 mg/kg of Asacol rectally
- Group IV (positive control 2) received 10 mg/kg of mesalazine orally
- Group V received 1 ml of 5% gel form of shark liver oil rectally
- Group VI received 1 ml of 10% gel form of shark liver oil rectally
- Group VII received 200 mg (0.22 ml) of shark liver oil orally
- Group VIII received 400 mg (0.44 ml) of shark liver oil orally

### 2.6. Weighing and Sampling

We recorded animal weights before the experiment (day 0) and at days of 1, 3, 5, and 7 using a digital scale with 0.1 g precision. Weight changes percentages were calculated using following formula:

(1) $$\begin{array}{c} \text{percentage of weight change}=\frac{\left({\text{weight}}_{\text{day}n}-{\text{weight}}_{\text{day}\left(n-1\right)}\right)}{{\text{weight}}_{\text{day}\left(n-1\right)}}\;\times 100. \end{array}$$

### 2.7. Histopathological Evaluations

All animals were sacrificed after seven days of treatment under deep ether anesthesia. Laparotomy was performed, and 8 cm of the distal of affected colon was excised and opened by longitudinal incision. After washing the tissue by normal saline, 6 cm of the specimen was fixed in 10% formaldehyde solution for histopathological evaluation. The remaining tissues were stored in liquid nitrogen till biochemical analysis. Formaldehyde-fixed tissues were embedded in paraffin. Blocks were divided into 5 *μ*m thick sections and stained with hematoxylin and eosin. We studied one slide for each animal. The representative slides for each animal contained both UC-affected and normal regions, were studied under light microscopic, and reviewed by a pathologist in a blinded fashion. The inflammation severity, inflammation extent, crypt damage, percentage of involvement, and regeneration are evaluated as given in Table 1 [24].

### 2.8. Oxidative Stress Evaluation

To biochemical analysis, 0.5 g of each frozen colonic tissue was mechanically homogenized in 5 ml of 0.05 M phosphate buffer saline pH 7.4 on ice to prevent heat shock. The homogenized samples were centrifuged (3500 rpm, 15 minutes, and 4°C), and their supernatants were collected and stored at −20°C for further evaluation of tissue total antioxidant capacity (TAC) by ELISA (ZB-TAC-96A, ZellBio GmbH, Germany) and activity of superoxide dismutase (SOD) by ELISA (ZB-SOD-96A, ZellBio GmbH, Germany) and glutathione peroxidase (GPx) by ELISA (BXC0551, Biorexfars, Iran).

### 2.9. Liver and Kidney Function Evaluation

At the end of the experiment, 2 ml of peripheral blood was collected into a vacutainer tube from each rat. Samples were centrifuged at 3000 rpm for 15 min at 4°C, and then, the serum was aliquoted and stored at a −80°C freezer. The serum concentration of aspartate aminotransferase (AST), alanine aminotransferase (ALT), alkaline phosphatase (ALP), albumin, total protein, indirect and total bilirubin, creatinine, and urea were detected by the autoanalyzer BT-3000 (Biotecnica Instruments, Italy) using commercial kits and reagents (Pars Azmoon Co., Iran).

### 2.10. Statistical Analysis

Data were expressed as mean and standard deviation (SD) or mean rank. SPSS version 21 for statistical analysis and GraphPad Prism 8.0 for drawing figure were used. The percentage of body weight change was analyzed by two-way ANOVA and Dunnett's multiple comparisons test. Antioxidant statuses and liver and kidney function tests were analyzed by one-way ANOVA and Tukey's post hoc test. Sum of histopathological scores in different groups were used as a cumulative scale which are presented in Table 2. The histopathological scores were analyzed using the nonparametric test of the Kruskal–Wallis *H* test. *P* values that are lesser than 0.05 were considered as a significant difference.

## 3. Results

### 3.1. Bodyweight

**Figure 1:**
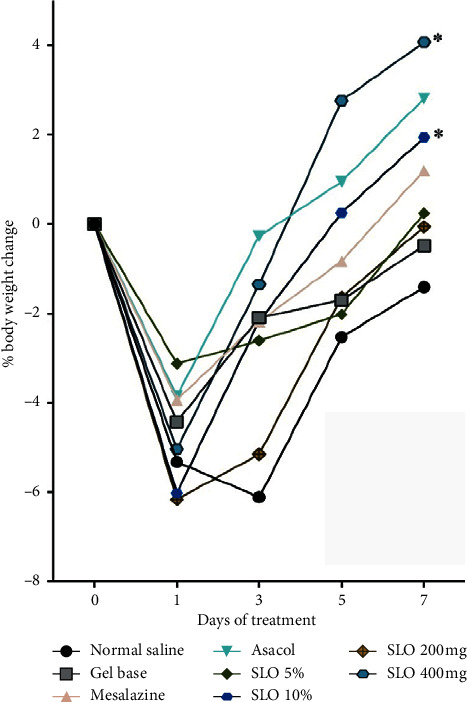
Effect of different treatments on acetic acid-induced rat's bodyweight change. Rats were treated with 1 ml normal saline, 1 ml gel base, 10 mg/kg mesalazine, 10 mg/kg Asacol, 5% rectal SLO, 10% rectal SLO, 200 mg oral SLO, or 400 mg oral SLO for seven days after UC induction. The percentage of weight gain at day 3, 5, and 7 were compared to day 1. Groups treated by 10% rectal SLO or 400 mg oral SLO had a significant weight gain on day 7, in comparison with day 1. The analysis was performed using two-way ANOVA and the multiple comparisons test of Dunnett. Data are presented as mean differences. ^*∗*^*P* < 0.05.

We restricted rat's access to food for 24 h before the experiment begins. All groups showed bodyweight loss due to the NPO time. On the 7^th^ day, all groups showed increased weight compared to day 1 of experiment. However, groups that treated by 10% SLO and 400 mg SLO presented a significant percentage of weight gain compared to day 1 (*P* value <0.05) (Figure 1).

### 3.2. Evaluation of Oxidative Stress Markers

**Figure 2:**
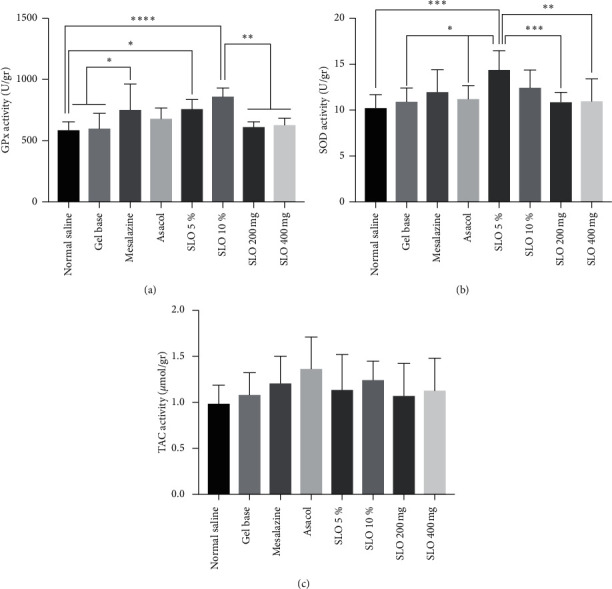
Effect of different treatment strategies on the biochemical assay of acetic acid-induced UC rats. Rats were sacrificed on day 7, and colon were dissected and homogenized and examined for GPx, SOD, and TAC. (a) Groups treated by 5% and 10% rectal SLO showed higher GPx activity. (b) SOD activity is significantly increased in the 5% SLO group. (c) No significant differences in TAC activity were detected among groups. The analysis was performed using one-way ANOVA and Tukey's post hoc test. Data are presented as mean ± SD. ^*∗*^*P* < 0.05, ^*∗∗*^*P* < 0.01, ^*∗∗∗*^*P* < 0.001, and ^*∗∗∗∗*^*P* < 0.0001.

As shown in Figure 2, GPx activity is significantly increased in 5% and 10% SLO groups compared to the normal saline group (*P* < 0.05 and *P* < 0.0001, respectively). In addition, 10% SLO-treated groups had a significantly higher GPx activity than gel base, Asacol, SLO 200 mg, and SLO 400 mg (*P* < 0.001, *P* < 0.05, *P* < 0.01, and *P* < 0.01, respectively).

According to our results, SOD activity of the group treated by 5% SLO is significantly elevated compared to normal saline, gel base, Asacol, SLO 200 mg, and SLO 400 mg (*P*=0.0001, *P* < 0.05, *P* < 0.05, *P* < 0.001, and *P* < 0.01). However, we observed no significant differences in TAC results among groups (*P* > 0.05).

### 3.3. Histopathological Examination and Microscopic Scoring for Ulcerative Colitis

**Figure 3:**
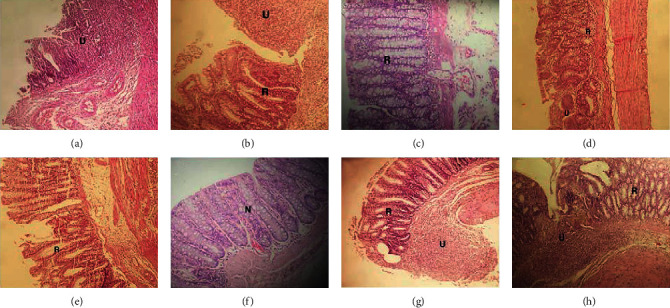
Histopathological features of colonic tissue biopsy specimens of ulcerative colitis-induced rats. (a) Normal saline: ulceration with mucosal regeneration, irregularity in crypt structure, goblet cell depletion, and inflammation of the mucosa. (b) Gel base: ulceration with mucosal regeneration and inflammation of the mucosa. (c) Asacol: near-complete regeneration of mucosa. (d) Mesalazine: ulceration with mucosal regeneration and inflammation of mucosa. (e) SLO 5%, rectal: ulceration with mucosal regeneration and inflammation of mucosa. (f) SLO 10%, rectal: near-complete regeneration of mucosa. (g) SLO 200 mg, oral: ulceration with adjacent mucosal regeneration. (h) SLO 400 mg, oral: focal surface ulceration with mucosal regeneration. H&E, ×100. N, normal tissue; R, regeneration; U, ulcer.

As shown in Figure 3, several histopathological abnormalities were observed due to acetic acid UC, induction including ulceration and loss of epithelial lining, goblet cell depletion, irregularity in crypt structure, and infiltration of inflammatory cells into colonic mucosa and submucosa. Certain histopathological features of colon tissues including inflammation severity, inflammation extent, crypt damage, percentage of involvement, and regeneration were evaluated.

Although we observed many signs of improvement including regeneration in Asacol and SLO groups, the mean rank of pathological changes did not represent a significant difference among groups (Table 1).

### 3.4. Liver and Kidney Function Evaluation

We examined creatinine, urea, AST, ALT, ALP, albumin, total protein, and indirect and total bilirubin levels to determine whether SLO treatments caused any kidney and liver dysfunctions or not. According to Table 3, a significant elevated AST level was detected in the group that received 400 mg of oral SLO compared to normal saline, gel base, and Asacol (*P* < 0.05). Our results showed no significant differences in other measured factors among groups.

## 4. Discussion

In this study, we focused on the antioxidant capacity of SLO and its effect on an acetic acid-induced ulcerative colitis in rat as a model. Our results showed that treating an animal model of UC by SLO can improve some aspects of oxidative stress and prevent the progress of the disease. Besides, SLO had significant positive effects on weight gain in 10% SLO and 400 mg SLO groups. However, no significant changes were observed in TAC activity or histopathological evaluations. Although it is possible that low dose and the short period of treatment were the reasons that we did not observe a significant improvement in colon tissue regeneration and other pathological aspects [25, 26]. To our knowledge, this is the first time that SLO has been evaluated for its potential application as a UC therapeutic agent in the rat animal model. Previously, Hünka et al. indicated that cod liver oil prevents oxidative stress and enhances glucose and lipid metabolism in diabetic rats [27]. Moreover, according to many studies, SLO seems to have biological activities including immunological defense enhancement, antitumor and antimetastasis activities, and antibacterial effects [28–30].

Ulcerative colitis is known as a chronic immune-mediated complication with an increasing number of affected patients around the world. The main goal of UC therapeutic strategies is to prolong the remission or corticosteroid-free period and reduce the risk of hospitalization and colorectal neoplasia [6]. UC management is mainly based on anti-inflammatory and immunosuppressant drugs, which have many adverse effects. Hence, there is an urgent need for better therapeutic approaches with high efficacy and lower side effects. Considering extensive research studies, oxidative stress has been proposed to play a crucial role in pathogenesis and exacerbation of this disease [31–33]. It has been documented that UC is associated with extensive amount of reactive oxygen species (ROS), which may be due to either overproduction or insufficient scavenging of ROS. The lack of balance between ROS production and antioxidant capacity may lead to oxidative stress in UC. Many researchers believe that the combination of antioxidant and anti-inflammatory natural agents could be a practical therapeutic approach for UC [34–37]. Shark liver oil has been known for its antioxidant and anti-inflammatory effects [27, 38]. SLO contains natural alkylglycerols and squalene, which are known for their immune modulatory and antioxidant activates, respectively [39].

Despite our results, this study has some limitations. First, since we did not assess the inflammatory markers, including interleukins and tissue necrosis factor, we cannot discuss the anti-inflammatory effect of SLO. Second, it is possible that the short period of treatment and low doses of SLO could be the reasons that we did not observe any significant improvement in the histopathological evaluations [25].

## Figures and Tables

**Table 1:** Histological grading of ulcerative colitis.

Scoring parameters	Score definition
Inflammation severity	0: none 1: mild 2: moderate 3: severe

Inflammation extent	0: none 1: mucosa 2: mucosa and submucosa 3: transmural

Crypt damage	0: none 1: basal 1/3 damaged 2: basal 2/3 damaged 3: crypts lost, surface epithelium intact 4: crypts lost, surface epithelium lost

Percentage of involvement	0: 0% 1: 1–25% 2: 26–50% 3: 51–75% 4: 76–100%

Regeneration	0: complete regeneration or normal tissue 1: almost complete regeneration 2: regeneration with crypt depletion 3: surface epithelium not intact 4: no tissue repair

**Table 2:** Comparison of histopathological scores among different groups.

	Normal saline	Gel base	Asacol	Mesalazine	SLO 5%	SLO 10%	SLO 200 mg	SLO 400 mg	*P* value
Inflammation severity	49.85	52.45	48.82	42.46	38.1	29	48.82	43.05	0.2484
Inflammation extent	50.65	52.27	49.36	43.33	38.4	32.11	47.55	45.4	0.3867
Crypt damage	44.8	50.95	48.09	49	39.95	30	48.09	43.6	0.5124
Percentage of involvement	54	51.73	51.05	46.58	38.4	30.83	47.5	38.83	0.1713
Regeneration	35.75	38.73	46.77	51.67	42.85	32.5	49.05	47.2	0.4778

Histopathological changes were evaluated according to Table 1.

**Table 3:** Effect of different treatments on kidney and liver function of acetic acid-induced rats.

Index	N/S	Gel base	Mesalazine	Asacol	SLO 5%	SLO 10%	SLO 200 mg	SLO 400 mg
Creatinine (mg/dl)	0.62 ± 0.034	0.45 ± 0.022	0.48 ± 0.033	0.54 ± 0.028	0.58 ± 0.064	0.63 ± 0.035	0.62 ± 0.078	0.45 ± 0.061
Urea (mg/dl)	22.60 ± 2.79	20.11 ± 2.42	17.77 ± 2.90	20.26 ± 1.36	21.39 ± 3.73	20.15 ± 2.14	23.21 ± 1.03	22.31 ± 1.06
AST (U/L)	115.6 ± 17.36	111.7 ± 16.06	124.6 ± 17.02	102.3 ± 18.32	130 ± 16.07	128.7 ± 13.29	129.2 ± 34.99	146.3 ± 30.11*∗*†^◉^
ALT (U/L)	77.3 ± 20.78	68.75 ± 8.17	59.4 ± 14.09	78.42 ± 10.11	63 ± 18.04	71.55 ± 8.57	77 ± 19.48	84 ± 16.44
ALP (U/L)	711.8 ± 210	726 ± 173	741 ± 149	754 ± 192	627 ± 104	618 ± 125	705 ± 246	733 ± 219
Albumin (g/dl)	3.15 ± 0.14	3.08 ± 0.11	3.11 ± 0.13	3.02 ± 0.11	3.14 ± 0.25	2.98 ± 0.23	3.32 ± 0.18	3.43 ± 0.18
Total protein (g/dl)	6.18 ± 0.38	5.81 ± 0.37	5.75 ± 0.25	5.98 ± 0.22	6.27 ± 0.58	6.04 ± 0.57	6.59 ± 0.11	6.71 ± 0.23
Indirect bilirubin (mg/dl)	0.09 ± 0.023	0.09 ± 0.005	0.08 ± 0.006	0.09 ± 0.006	0.1 ± 0.006	0.09 ± 0.009	0.04 ± 0.08	0.04 ± 0.29
Total bilirubin (mg/dl)	0.13 ± 0.013	0.12 ± 0.019	0.13 ± 0.011	0.13 ± 0.008	0.13 ± 0.003	0.12 ± 0.043	0.06 ± 0.011	0.08 ± 0.036

The analysis was performed using one-way ANOVA and Tukey's post hoc test. Data are presented as mean ± SD. ^*∗*^SLO vs. normal saline; ^✝^SLO vs. gel base; ^◉^SLO vs. Asacol.

## Data Availability

The data used to support the findings of this study are available from the corresponding author upon reasonable request.

## References

[1] Hindryckx P., Jairath V., D’Haens G. (2016). Acute severe ulcerative colitis: from pathophysiology to clinical management. *Nature Reviews Gastroenterology & Hepatology*.

[2] Ananthakrishnan A. N. (2015). Epidemiology and risk factors for IBD. *Nature Reviews Gastroenterology & Hepatology*.

[3] Kasper D., Fauci A., Hauser S., Longo D., Jameson J., Loscalzo J. (2015). *Harrison’s Principles of Internal Medicine, 19e*.

[4] Eaden J. A., Abrams K. R., Mayberry J. F. (2001). The risk of colorectal cancer in ulcerative colitis: a meta-analysis. *Gut*.

[5] Ahluwalia B., Moraes L., Magnusson M. K., Öhman L. (2018). Immunopathogenesis of inflammatory bowel disease and mechanisms of biological therapies. *Scandinavian Journal of Gastroenterology*.

[6] Rubin D. T., Ananthakrishnan A. N., Siegel C. A., Sauer B. G., Long M. D. (2019). ACG clinical guideline: ulcerative colitis in adults. *The American Journal of Gastroenterology*.

[7] Alulis S., Vadstrup K., Borsi A. (2020). Treatment patterns for biologics in ulcerative colitis and Crohn’s disease: a Danish nationwide register study from 2003 to 2015. *Scandinavian Journal of Gastroenterology*.

[8] Poggioli G., Gentilini L., Coscia M., Boschi L., Ugolini F. (2019). Evolution of surgical treatment of ulcerative colitis. *Ulcerative Colitis*.

[9] Alatab S., Sepanlou S. G., Ikuta K. (2020). The global, regional, and national burden of inflammatory bowel disease in 195 countries and territories, 1990–2017: a systematic analysis for the global burden of disease study 2017. *The Lancet Gastroenterology & Hepatology*.

[10] Vadstrup K., Alulis S., Borsi A. (2020). Societal costs attributable to Crohn’s disease and ulcerative colitis within the first 5 years after diagnosis: a Danish nationwide cost-of-illness study 2002–2016. *Scandinavian Journal of Gastroenterology*.

[11] Krotkiewski M., Przybyszewska M., Janik P. (2003). Cytostatic and cytotoxic effects of alkylglycerols (Ecomer). *Medical Science Monitor: International Medical Journal of Experimental and Clinical Research*.

[12] Skopińska-Rózewska E., Chorostowska-Wynimko J., Krotkiewski M. (2003). Inhibitory effect of Greenland shark liver oil combined with squalen and arctic birch ashes on angiogenesis and L-1 sarcoma growth in Balb/c mice. *Polish Journal of Veterinary Sciences*.

[13] Lewkowicz P., Banasik M., Glowacka E., Lewkowicz N., Tchorzewski H. (2005). Effect of high doses of shark liver oil supplementation on T cell polarization and peripheral blood polymorphonuclear cell function. *Polski Merkuriusz Lekarski*.

[14] Vázquez L., Fornari T., Señoráns F. J., Reglero G., Torres C. F. (2008). Supercritical carbon dioxide fractionation of nonesterified alkoxyglycerols obtained from shark liver oil. *Journal of Agricultural and Food Chemistry*.

[15] Pugliese P. T., Jordan K., Cederberg H., Brohult J. (1998). Some biological actions of alkylglycerols from shark liver oil. *The Journal of Alternative and Complementary Medicine*.

[16] Palmblad J., Samuelsson J., Brohult J. (1990). Interactions between alkylglycerols and human neutrophil granulocytes. *Scandinavian Journal of Clinical and Laboratory Investigation*.

[17] Osmond D. G., Roylance P. J., Webb A. J., Yoffey J. M. (1963). The action of batyl alcohol and selachyl alcohol on the one marrow of the Guinea pig. *Acta Haematologica*.

[18] Pedrono F., Martin B., Leduc C. (2004). Natural alkylglycerols restrain growth and metastasis of grafted tumors in mice. *Nutrition and Cancer*.

[19] Skopinska-Rozewska E., Krotkiewski M., Sommer E. (1999). Inhibitory effect of shark liver oil on cutaneous angiogenesis induced in Balb/c mice by syngeneic sarcoma L-1, human urinary bladder and human kidney tumour cells. *Oncology Reports*.

[20] Brohult A., Brohult J., Brohult S., Joelsson I. (1979). Effect of alkoxyglycerols on the frequency of fistulas following radiation therapy for carcinoma of the uterine cervix. *Acta Obstetricia et Gynecologica Scandinavica*.

[21] Association WM (2008). *World Medical Association Declaration of Helsinki: Ethical Principles for Medical Research Involving Human Subjects*.

[22] Sunarya, Hole M., Taylor K. A. (1996). Methods of extraction composition and stability of vitamin A and other components in dogfish (*Squalus acanthias*) liver oil. *Food Chemistry*.

[23] Millar A. D., Rampton D. S., Chander C. L. (1996). Evaluating the antioxidant potential of new treatments for inflammatory bowel disease using a rat model of colitis. *Gut*.

[24] Dieleman L. A., Palmen M. J., Akol H. (1998). Chronic experimental colitis induced by dextran sulphate sodium (DSS) is characterized by Th1 and Th2 cytokines. *Clinical & Experimental Immunology*.

[25] Samimi N., Farjam M. (2019). The effect of cupressus sempervirens on ulcerative colitis: do pathological changes improve by oxidative stress amelioration?. *Galen Medical Journal*.

[26] Sepehrimanesh M., Samimi N., Koohi-Hosseinabadi O., Mokhtari M., Amiri-Zadeh S., Farjam M. (2018). Effects of *Cupressus sempervirens* extract on the healing of acetic acid-induced ulcerative colitis in rat. *Journal of Coloproctology*.

[27] Hünkar T., Aktan F., Ceylan A., Karasu Ç. (2002). Effects of cod liver oil on tissue antioxidant pathways in normal and streptozotocin-diabetic rats. *Cell Biochemistry and Function*.

[28] Iagher F., de Brito Belo S. R., Naliwaiko K. (2011). Chronic supplementation with shark liver oil for reducing tumor growth and cachexia in walker 256 tumor-bearing rats. *Nutrition and Cancer*.

[29] Hajimoradi M., Hassan Z. M., Pourfathollah A. A., Daneshmandi S., Pakravan N. (2009). The effect of shark liver oil on the tumor infiltrating lymphocytes and cytokine pattern in mice. *Journal of Ethnopharmacology*.

[30] Lewkowicz P., Banasik M., Głowacka E., Lewkowicz N., Tchorzewski H. (2005). Effect of high doses of shark liver oil supplementation on T cell polarization and peripheral blood polymorphonuclear cell function. *Polski Merkuriusz Lekarski: Organ Polskiego Towarzystwa Lekarskiego*.

[31] Piechota-Polanczyk A., Fichna J. (2014). Review article: the role of oxidative stress in pathogenesis and treatment of inflammatory bowel diseases. *Naunyn-Schmiedeberg’s Archives of Pharmacology*.

[32] Rezaie A., Parker R. D., Abdollahi M. (2007). Oxidative stress and pathogenesis of inflammatory bowel disease: an epiphenomenon or the cause?. *Digestive Diseases and Sciences*.

[33] Uraz S., Tahan G., Aytekin H., Tahan V. (2013). *N*-acetylcysteine expresses powerful anti-inflammatory and antioxidant activities resulting in complete improvement of acetic acid-induced colitis in rats. *Scandinavian Journal of Clinical and Laboratory Investigation*.

[34] Balmus I., Ciobica A., Trifan A., Stanciu C. (2016). The implications of oxidative stress and antioxidant therapies in inflammatory bowel disease: clinical aspects and animal models. *Saudi Journal of Gastroenterology*.

[35] Si-Yu C., Sheng-Jie Y., Wei-Wei W., Bing W., Zhang T., Yi-Qiong P. (2019). Progress in active compounds effective on ulcerative colitis from Chinese medicines. *Chinese Journal of Natural Medicines*.

[36] Asakura H., Kitahora T. (2018). *Antioxidants and Polyphenols in Inflammatory Bowel Disease: Ulcerative Colitis and Crohn Disease. Polyphenols: Prevention and Treatment of Human Disease*.

[37] Lankarani K. B., Sepehrimanesh M., Seghatoleslam S. F., Hoseini S. E., Ghavami S. (2017). Autophagy-related protein 7 level in patients with ulcerative colitis. *Scandinavian Journal of Gastroenterology*.

[38] Deniau A.-L., Mosset P., Pédrono F., Mitre R., Bot D. L., Legrand A. B. (2010). Multiple beneficial health effects of natural alkylglycerols from shark liver oil. *Marine Drugs*.

[39] Vadalà M., Laurino C., Palmieri L., Palmieri B. (2017). Shark derivatives (Alkylglycerols, Squalene, Cartilage) as putative nutraceuticals in oncology. *European Journal of Oncology*.

